# Carbon Supported Engineering NiCo_2_O_4_ Hybrid Nanofibers with Enhanced Electrocatalytic Activity for Oxygen Reduction Reaction

**DOI:** 10.3390/ma9090759

**Published:** 2016-09-06

**Authors:** Diab Hassan, Sherif El-safty, Khalil Abdelrazek Khalil, Montasser Dewidar, Gamal Abu El-magd

**Affiliations:** 1 Mechanical Design and Materials Department, Faculty of Energy Engineering, Aswan University, Aswan 81521, Egypt; diab_khalaf@aswu.edu.eg; 2 National Institute for Materials Science (NIMS), Research Center for Strategic Materials, 1-2-1Sengen, Tsukuba-shi, Ibaraki-ken 305-0047, Japan; Sherif.ELSAFTY@nims.go.jp; 3 Graduate School of Advanced Science and Engineering, Waseda University, 3-4-1 Okubo, Shinjuku-Ku, Tokyo 169-8555, Japan; 4 Department of Mechanical Engineering, College of Engineering, King Saud University, P.O. Box 800, Riyadh 11421, Saudi Arabia; 5 Department of Mechanical Engineering, Faculty of Engineering, Kafrelsheikh University, Elgaishstreet, Kafrelsheikh 33516, Egypt; dewidar5@hotmail.com; 6 Production Engineering and Design Department, Faculty of Engineering, Minia University, El-Minia 61519, Egypt; g_magd@mu.edu.eg

**Keywords:** NiCo_2_O_4_, PAN, electrospinning, nanofiber, ORR

## Abstract

The design of cheap and efficient oxygen reduction reaction (ORR) electrocatalysts is of a significant importance in sustainable and renewable energy technologies. Therefore, ORR catalysts with superb electrocatalytic activity and durability are becoming a necessity but still remain challenging. Herein, we report C/NiCo_2_O_4_ nanocomposite fibers fabricated by a straightforward electrospinning technique followed by a simple sintering process as a promising ORR electrocatalyst in alkaline condition. The mixed-valence oxide can offer numerous accessible active sites. In addition, the as-obtained C/NiCo_2_O_4_ hybrid reveals significantly remarkable electrocatalytic performance with a highly positive onset potential of 0.65 V, which is only 50 mV lower than that of commercially available Pt/C catalysts. The analyses indicate that C/NiCo_2_O_4_ catalyst can catalyze O_2_-molecules via direct four electron pathway in a similar behavior as commercial Pt/C catalysts dose. Compared to single NiCo_2_O_4_ and carbon free NiCo_2_O_4_, the C/NiCo_2_O_4_ hybrid displays higher ORR current and more positive half-wave potential. The incorporated carbon matrices are beneficial for fast electron transfer and can significantly impose an outstanding contribution to the electrocatalytic activity. Results indicate that the synthetic strategy hold a potential as efficient route to fabricate highly active nanostructures for practical use in energy technologies.

## 1. Introduction

The depletion of natural fossil fuels and tremendous growth in environmental pollution have attracted extensive interests from concerned individuals and governments. Exploring alternative energy systems with high efficiency is of great importance to meet the needs of modern society and global ecological concerns [1,2].

Fuel cells such as polymer electrolyte membrane fuel cells (PEMFCs) and direct methanol fuel cells (DMFC) are of significant importance to substitute or even diminish the utilization of commercially available fossil fuel [3,4,5]. These devices show preferable features including high power density and zero emission. However, developing highly efficient and cost-effective energy storage or conversion devices remains a great challenge. 

Oxygen reduction reaction (ORR) plays an important role in renewable energy systems such as fuel cells and batteries [6]. Accordingly, it is a universal cathode reaction which can reduces the oxygen molecules to water and can be achieved via a direct four electron pathways [7,8]. The highly active platinum-based electrocatalysts are known as the most effective ORR catalysts [9,10]. However, their high cost, scarcity and sluggish ORR kinetics have prompted a recent drive towards the synthesis of cost-effective and high-performance non-precious ORR electrocatalysts [11]. The current bottleneck for improving energy technologies (i.e., fuel cells and air batteries) is the electroactive material which can remarkably affect the overall device performance. Various electrocatalytic materials have been investigated with various successes [12,13].

The development of highly active and stable electrocatalysts with unique ORR features is gradually becoming of paramount significance. A great deal of attention has been focused on the design and synthesis of inexpensive catalysts prepared mainly from earth-abundant components.

To date, the earth-abundant transition metal oxides based materials as electrocatalysts are gaining generous interest due to their broad applicability in clean energy technologies like fuel cells and metal–air batteries [14,15] due to their attractive features of low preparation cost, considerably high catalytic properties, and superior electrochemical stability.

Cobalt oxides based materials having superb structural and compositional semblance with enriched electroactive sites are considered as feasible candidate for ORR [16,17,18]. Hybridizing two metal oxides has been considered as potential class of alternatives that can significantly boost the electrochemical performance towards ORR [19,20]. Among the new alternatives, cobalt-nickel based oxides exhibit higher electrocatalytic activity than single cobalt oxides or nickel oxides due to their mixed valences which facilitate the electron/ion transportation and redox reactions [21,22,23].

Subsequently, nickel cobaltite (NiCo_2_O_4_) nanostructures have been widely investigated as electrode materials in the field of electrochemical supercapacitor [24], Li-ion batteries [25,26], and chemical sensors [27], and direct alcohols fuel cells [28,29]. For example, Prathap et al. [30] demonstrated that the urchin-like NiCo_2_O_4_ fabricated by a straightforward hydrothermal method had excellent electroactivity for methanol electrooxidation in alkaline solution. Zhang and coworkers reported NiCo_2_O_4_/N-rGO hybrid with improved catalytic performance for ORR close to that of commercial carbon-supported Pt and an onset potential of −0.12 V [31]. In addition, Liu et al. prepared NiCo_2_O_4_@ZnCo_2_O_4_ core−sheath nanowires with much enhanced electrocatalytic activity for the ORR [20].

Undoubtedly, the development of a simple, low cost and, scalable synthesis strategy to prepare catalytically active hybrids with a controlled surface structure and composition becomes the focal task. The recently reported literature demonstrated that the catalytic reactivity of nanostructured materials can be effectively enhanced by structure manipulation of materials [32,33].

However, conventional synthesis approaches suffer from many disadvantages such as complex procedure, high cost, and limited applicability. Therefore, it will be of great importance to adapt a facile and cost effective fabrication route which can be extended to successfully prepare efficient ORR hybrid catalysts at high yield. Additionally, porous nanostructures can efficiently decrease the resistance of mass transported and facilitate the transfer of reactants species to the catalytically active sites, thus is significantly preferable for electrochemical reactions [34].

Different synthesis strategies have been investigated for the preparation of NiCo_2_O_4_ materials including electrostatic spray deposition [35], chemical deposition [28], spray pyrolysis [36,37], and dipping printing deposition [38]. Compared to traditional synthesis routes, electrospinning is simple, straightforward, and powerful technique which can be utilized to produce one dimensional (1-D) nanostructures with high surface area at diameters ranging from several hundred to tens of nanometers [39,40,41,42,43]. As an efficient technique, electrospinning opened up a new avenue for the fabrication of nanosized materials for ORR [44].

NiCo_2_O_4_ nanostructures enjoy a place of pride owing to their favored features. However, the ORR reactivity of single NiCo_2_O_4_ is strongly affected by its low electrical conductivity and relatively limited active sites. To tackle these issues and achieve much higher electrochemical performance for ORR, the key solution is to integrate NiCo_2_O_4_ with highly conductive materials (i.e., graphene, carbon, etc.) to efficiently improve the electronic configuration and mobility of transferred electrons. Recent studies indicated that combining NiCo_2_O_4_ with graphene counterparts can greatly boost the ORR activity due to fast electron transportation and synergetic effect of NiCo_2_O_4_ and graphene [8,31].

The main target of the present work is to change this by providing conspicuous advancements. This study unravels the mechanistic key role of redox-active metal cations and carbon matrices in improving the ORR of the obtained hybrid which might open new opportunities for designing highly active electrocatalysts.

On the basis of the aforementioned consideration, carbon supported nickel cobaltite nanofibers denoted as C/NiCo_2_O_4_ were developed via a simple and scalable electrospinning method followed by an annealing treatment at high temperature. The as-synthesized composite was utilized as a promising ORR catalyst. Benefitting from the elegant structural features of 1-D mesoporous structure, homogenous physical/chemical interaction at the nanoscale level, and strong coupling effect, the as-obtained C/NiCo_2_O_4_ hybrid nanofibers presents significantly higher ORR electrocatalytic activity than single NiCo_2_O_4_ and carbon-free NiCo_2_O_4_. C/NiCo_2_O_4_ exhibits high cathodic current very close to that of commercial Pt/C and superior electrochemical durability. These findings are mainly attributed to accessible active sites, synergetic effect of both metallic species (Co and Ni species) and counterparts, improved conductivity, and fast electron transport. Thus greatly enhance ORR electrocatalytic performance.

Results manifested that the mesoporous C/NiCo_2_O_4_ nanofibers fabricated by electrospinning method can be potentially applied in high performance energy conversion or storage systems.

## 2. Materials and Methods

### 2.1. Materials

Cobalt (II) acetate tetrahydrate (Co(CH_3_COO)_2_·4H_2_O, CoAc) and nickel (II) acetate tetrahydrate (Ni(CH_3_COO)_2_·4H_2_O, NiAc) were supplied from wako.co, Osaka, Japan. Polyacrylonitrile (PAN, Mw = 150,000) and *N*,*N*-dimethylformamide (DMF, ≥99.5%) were supplied by Sigma-Aldrich Company Ltd., St. Louis, MO, USA. All the investigated chemicals and reagents were directly used without further purification.

### 2.2. Preparation of C/NiCo_2_O_4_ and NiCo_2_O_4_ Nanofibers by Electrospinning Method

The C/NiCo_2_O_4_ nanofibers were successfully synthesized by a facile electrospinning technique followed by two subsequent heat treatments. To prepare the solution, 0.7 g of CoAc and 0.35 g of NiAc were added to 10 g of DMF under magnetic stirring at room temperature for at least 4 h to form a transparent solution. Another solution was prepared by dissolving 0.25 g of PAN in 8 g of DMF followed by vigorous mechanical stirring for 3 h at 70 °C and then cooled to room temperature. The precursor solutions were then mixed and the resulting mixture was continuously stirred until a homogeneous solution formed. Next, the as-prepared mixture was loaded into a plastic syringe (10 mL) connected to a stainless steel needle (~0.3 mm inner diameter). The feeding rates of the electrospinning solution was controlled using a digital pump. A rectangular metal plate wrapped by thin aluminum foil was served as a collector. The distance between the needle tip and collector was maintained at 15 cm. Then, the as-obtained solution was electrospun with an applied voltage of 15 kV. The as-spun mats were carefully peeled off from the aluminum foil and dried under vacuum at 80 °C for 10 h. The dried mats were first stabilized in an air atmosphere at 250 °C for 2 h and then annealed at 600 °C under argon flow for 4 h using a horizontal tube furnace with a heating rate of 5 °C min^−1^ to produce the final porous C/NiCo_2_O_4_. For comparison, NiCo_2_O_4_ nanofibers were fabricated by same procedure using CoAc and NiAc precursors in the absence of PAN and the stabilized fibers were calcined at 400 °C for 3 h in open air at 5 °C min^−1^ heating ramp. 

### 2.3. Electrochemical Measurements

The electrochemical properties of C/NiCo_2_O_4_ nanofibers were collected in a conventional three-electrode system. An Ag/AgCl electrode filled with saturated KCl solution and Pt-wire were used as the reference and counter electrode, respectively. The electrocatalytic activities for ORR were analyzed in O_2_ saturated 0.1 M KOH solution. The solution was first purged with oxygen gas for at least 30 min before the experiment. To ensure O_2_-saturated electrolyte, the oxygen flow was kept above the solution during the electrochemical test. The working electrode was prepared by dissolving 5 mg of the synthesized C/NiCo_2_O_4_ nanofibers in 5 mL of de-ionized water under sonication for 30 min. Eight microliters of the as-prepared suspension was poured onto a glassy carbon electrode (GC) (3 mm diameter, 0.07065 cm^2^) followed by 30 µL (5 wt %) Nafion solution and then carefully dried to form a stable film of the active catalyst.

The commercially available Pt/C catalyst (20 wt % Pt, Alpha Aesar, Haverhill, MA, USA) was prepared by same protocol on GC. Cyclic voltammetry (CV), linear-sweep voltammograms (LSVs), electrochemical impedance spectroscopy (EIS), and chronoamperometry spectra (CA) were carried out on a Zennium/ZAHNER (Elektrik GmbH & Co. KG, Bisingen, Germany) electrochemical station. The LSV curves were performed on a rotating disk electrode (RDE, 5 mm diameter, 0.196 cm^2^) at a rotational speed of 1600 rpm. The current–time (i–t) characteristics of the catalysts were measured by chronoamperometry technique at a set potential 0.2 V (vs. Ag/AgCl) for 10,000 s in O_2_-saturated 0.1 M KOH solution. The Koutecky–Levich (K–L) equation [45,46] was investigated to estimate the number of electron transferred (*n*) per O_2_-molecules as follow:

(1) $$\frac{1}{J}=\frac{1}{J_{K}}+\frac{1}{{\beta \omega }^{0.5}}$$

where J is the diffusion-limited current density, $J_{K}$ is the kinetic current density, and ω is the rotational speed of the electrode given in rad·s^−1^. β is the Koutecky–Levich constant and can be measured from the slope of the K–L plots according to the equation.

(2) $$\beta =0.62nFC_{o}{D_{o}}^{2/3}\vartheta^{-1/6}$$

$D_{o}$ is the diffusion coefficient of O_2_ molecules in the solution (1.9 × 10^−5^ cm^2^ s^−1^), $C_{o}$ is the concentration of the oxygen molecules in the solution (1.2 × 10^−3^ mol cm^−3^), F is the Faradic constant (96,486 C mol^−1^), and ϑ is the kinematic viscosity of the solution (0.01 cm^2^ s^−1^).

### 2.4. Characterization of the Catalysts

The size and morphologies of the as-synthesized fibers were analyzed using field emission scanning electron microscopy (FE-SEM, Model 6500, JEOL, Peabody, MA, USA) at an acceleration voltage of 12 kV. Transmission electron microscopy (TEM, H-8100, Hitachi, Tokyo, Japan) operated at an acceleration voltage of 200 kV was employed to provide further the surface structure of the calcined product. The composition and phase purity of the samples were measured by wide angle—X-ray diffraction (WA-XRD, Bruker D8 Advance, Bruker Co., Spring, TX, USA) with CuKα-X-radiation (λ = 1.542 Å). Raman spectroscopy measurements were conducted on Horiba system (JobinYvon) using a laser excitation of 633 nm. The chemical compositions of the sample were obtained by X-ray photoelectron spectroscopy (XPS) using a ESCALAB250 spectrometer (Thermo Fisher Scientific corporation, Paisley, UK) equipped with AlKa radiation (hv = 1486.6 eV). The Brunauer–Emmett–Teller (BET) surface area and pore size distribution of the samples were determined by a BELSORP36 analyzer (JP. BEL Co., Ltd., Osaka, Japan) at 77 K. Before physisorption test, the samples were thermally pre-treated with purified N_2_ gas for 6 h.

## 3. Results and Discussion

### 3.1. Synthesis

**Figure 1:**
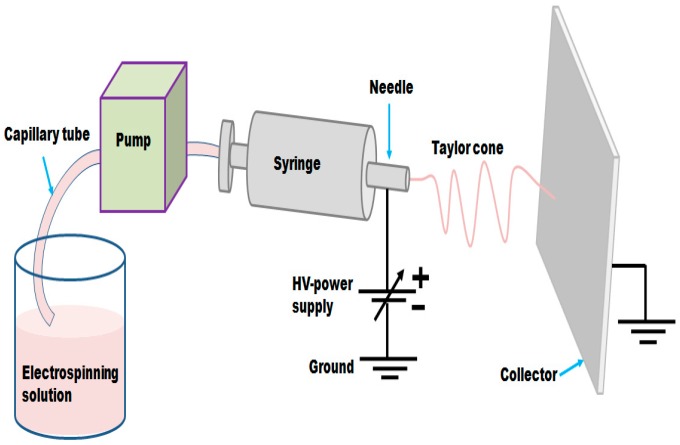
Graphical configuration illustrates the home made electrospinning technique applied for the synthesis of C/NiCo_2_O_4_ hybrid nanofibers. The mixed precursors were loaded into a plastic syringe using through a simple pumping system. When a high voltage of 15 kV was applied, the electrospinning solution moves forming a very thin mat of fibers on aluminum foil which surrounded the rectangular collector.

We have developed a simple synthesis route to fabricate nonwoven nanofibers using electrospinning method followed by two-step heat treatment as schematically illustrated in Figure 1. To achieve this, a homogeneous electrospun solution mainly composed of Ni acetate, Co acetate, and PAN was prepared. The as-prepared solution was then electrospun with the assistance of high voltage power supply which generates a high electrical potential (15 kV) between the needle tip and collector within a pre-set distance (15 cm) to produce 1-D highly interconnected and ultra-long nanofibers. The final products were obtained after two subsequent thermal treatments. In details, the as-spun fibers were stabilized in air at 250 °C for 2 h before cooling to room temperature. After that, the stabilized and then underwent a calcination process at 600 °C for 4 h under argon atmosphere. However, the calcination process has no effect on the fibrous nature of the fibers.

### 3.2. Morphology and Structure Analyses

**Figure 2:**
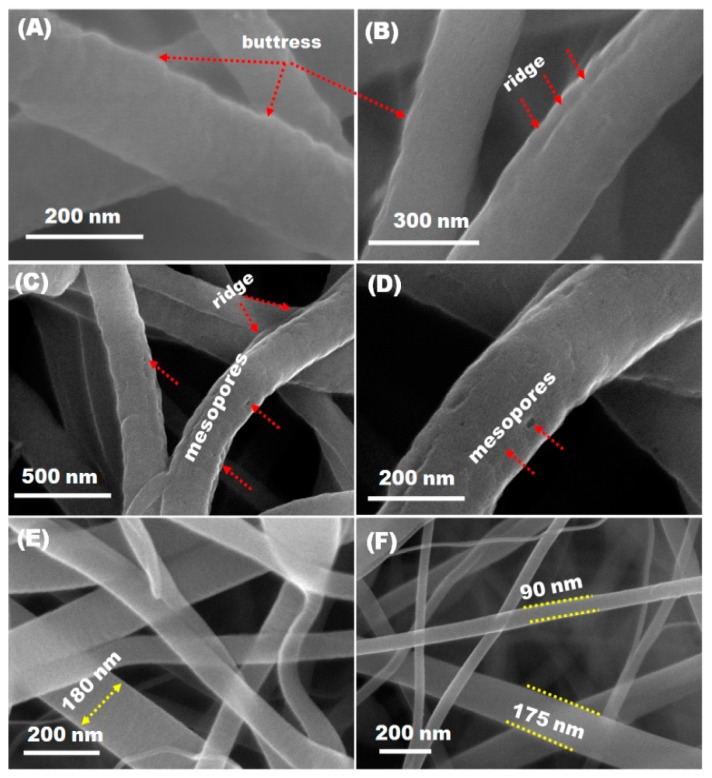
(**A**–**F**) Top-view FE-SEM micrographs of stabilized and calcined fibers measured at different locations with different magnifications: (**A**,**B**) low magnification SEM micrographs of stabilized NiCo_2_O_4_ nanofibers; and (**C**,**D**) low magnification SEM images of stabilized C/NiCo_2_O_4_ nanofibers. (**A**–**F**) SEM images of calcined nanofibers; (**E**) C/NiCo_2_O_4_; and (**F**) NiCo_2_O_4_. The red arrows indicate the buttress and ridges formed at the surface of the stabilized fibers (**A**,**B**); and show the generated mesopores in the mesoporous C/NiCo_2_O_4_ hybrid (**C**,**D**).

The morphological characteristics of the synthesized fibers were first investigated using field-emission scanning electron microscopy (FE-SEM). Figure S1A−D and Figure 2A−F show SEM images of as-electrospun (Figure S1A−D), stabilized, and calcined nanofibers. The fibers exhibit 1-D structures with a diameter sizes ranging from 200–250 nm (Figure 2A,B). Clearly, the doping of PAN ions does not affect the structure of the as-spun nanofibers. As a result, the size of the fibers decreased after thermal treatment whilst maintaining the 1-D structure. After carbonization process, the were transformed into carbon structure. As shown, the micrographs display randomly packed nanofibers, cross linked with each other which is beneficial for fast ion and electron diffusion [47]. In addition, the high-magnification SEM micrographs indicate that the stabilized nanofibers have rough surfaces with nanosized pores of 30–70 nm as indicated by the arrows in Figure 2C,D. These mesopores might be due to the outward release of solvent molecules and decomposition of outer metal salts during the heat treatment process. The diameter of the annealed nanofibers (Figure 2E,F) shrank drastically due to successful transformation of metal precursors to bi-component phase at peak temperature and thermal decomposition of PAN [48,49,50].

**Figure 3:**
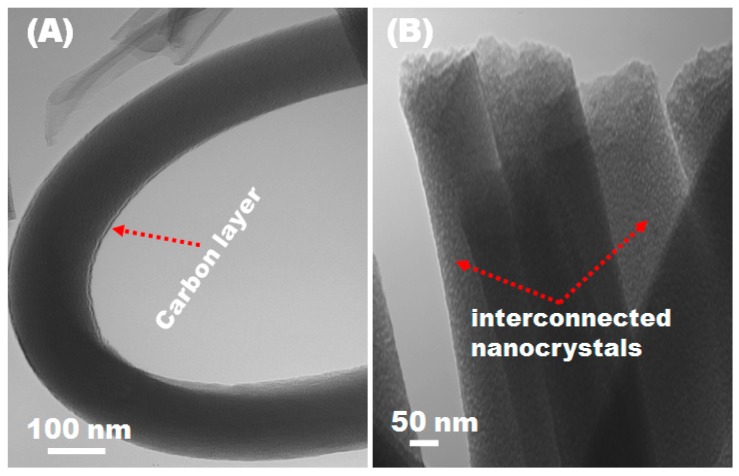
(**A**,**B**) TEM images of hierarchical mesoporousC/NiCo_2_O_4_ hybrid nanofibers show the surface morphology.

Transmission electron microscopy (TEM) analysis was carried out to provide further insight into the microstructure and morphological features of the porous C/NiCo_2_O_4_ nanofibers (Figure 3A,B). As clearly seen, compact nanofibers with quite smooth surfaces are obtained. It is interesting to observe that that the resultant fibers possess a well-defined mesoporous which can be attributed to the removal of organic moieties from the metallic precursors and polymer matrix. The morphology of the fibers was well preserved after sintering at 600 °C with a notable decrease in the average diameter which could be ascribed to the weight loss due to the decomposition of fibers at high temperature which in good agreement with the SEM observations.

Interestingly, the TEM observation clearly illustrate the formation of nanosized particle-by-particle ornamentation as a continuous 1-D building along the fiber direction. This is mainly attributed to the mixed metallic nanoparticles from which the fiber formed.

### 3.3. Crystallographic and Chemical Composition of Synthesized Nanofibers

To clarify the phase structure of the final products, XRD analysis was conducted as presented in Figure S2. As shown, the XRD patterns of the synthesized NiCo_2_O_4_ and C/NiCo_2_O_4_ nanofibers exhibited eight well-defined diffraction peaks corresponding to (111), (220), (311), (222), (400), (511), (440), and (533) planes that match well with to the standard profiles of the spinel NiCo_2_O_4_ phase (JCPDF card: 20-0781) [51]. The weak diffraction peak observed at 25° is mainly assigned to (002) plane of carbon. These results indicate that the precursor salts have been completely transformed to into NiCo_2_O_4_ at after thermal treatment. No other peaks are detected in the XRD patterns demonstrating the purity of the annealed powder.

**Figure 4:**
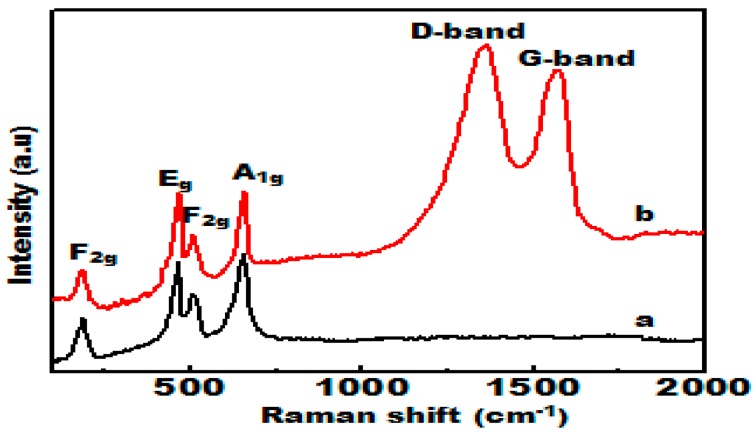
Raman spectra of (**a**) NiCo_2_O_4_ nanofibers and (**b**) C/NiCo_2_O_4_ hybrid nanofibers.

To further illustrate the chemical composition of the annealed samples, Raman spectroscopy analysis was performed. As observed in Figure 4, the Raman spectra of the NiCo_2_O_4_ and C/NiCo_2_O_4_ products reveal four prominent peaks located at 186, 464.6, 507.7 and 654.5 cm^−1^ assigned to the F_2g_, E_g_, F_2g_ and A_1g_ vibrational modes of spinel NiCo_2_O_4_, respectively [52,53]. The intense diffraction bands detected nearly at 1357 and 1566 cm^−1^ of the C/NiCo_2_O_4_ spectrum were due to the D and G bands of carbon, respectively. These findings match well with previously reported literature [54].

### 3.4. Surface Area and Porous Structure Investigation

**Figure 5:**
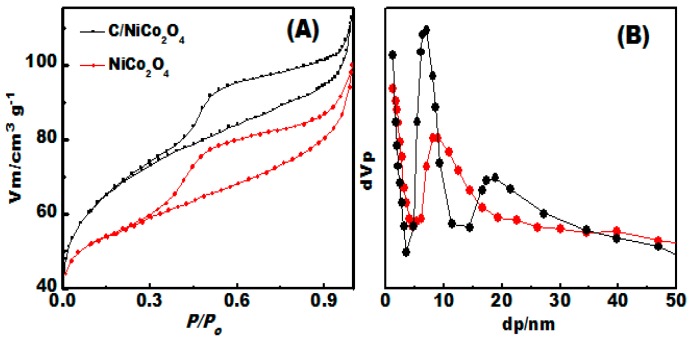
(**A**,**B**) Surface area and porous structure analyses: (**A**) N_2_-adsorption/desorption isotherms collected at 77 K; and (**B**) corresponding pore size distribution curves measured by NLDFT approach.

To check the porous structure, N_2_ adsorption–desorption isotherms C/NiCo_2_O_4_ and NiCo_2_O_4_ products were measured as given in (Figure 5A,B). The specific surface areas (S_BET_) of C/NiCo_2_O_4_ and NiCo_2_O_4_ were measured to be 123.9 and 94.6 m^2^ g^−1^ (Figure 5A), which is much higher than that those of previouslyreported metal oxide based catalysts [43]. This result indicate that the 1-D nanofibers can provide a high surface area. In addition, the pore size distribution (Figure 5B) for C/NiCo_2_O_4_ measured by NLFDT method exhibits a narrow distribution of mesopores with sizes ranging from 6.6 to 18.5 nm indicating a well-developed mesoporous structure. As a comparison, single NiCo_2_O_4_ shows a pore size distribution mainly centered at 8.7 nm.

Interestingly, the high specific surface area of C/NiCo_2_O_4_ is expected to enhance the contact area at the electrolyte/electrode interfaces, provide abundant active sites for electrochemical reaction. Furthermore, the unique porous structure can significantly facilitate the transport of ions and electrons into the pores and thus improve the electrochemical performance.

### 3.5. ORR Electrocatalytic Activity

**Figure 6:**
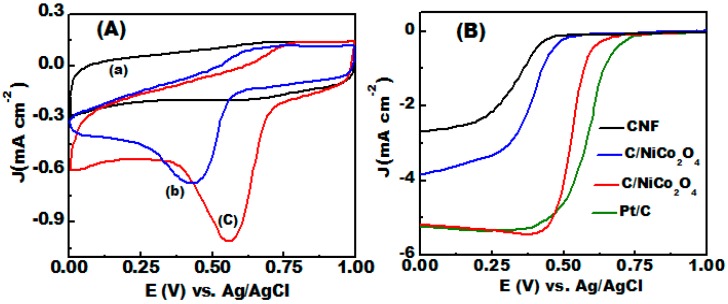
ORR electrocatalytic performances of the synthesized catalysts measured in N_2_ or O_2_-saturated 0.1 M KOH solutions: (**A**) CVs of the catalysts obtained at 50 mVs^−1^ scan rates at room temperature of (a) C/NiCo_2_O_4_ in N_2_-saturated solution (b) NiCo_2_O_4_ in O_2_-saturated solution and (c) C/NiCo_2_O_4_ in O_2_-saturated solution; and (**B**) LSVs responses of the prepared catalysts recorded at 1600 rpm compared with commercial Pt/C catalyst.

The electrocatalytic activity for ORR cyclic voltammograms of the as-prepared NiCo_2_O_4_ and C/NiCo_2_O_4_ nanofibers were measured in O_2_-saturated 0.1 M KOH solution at 50 mVs^−1^ at room temperature as presented in Figure 6A. A homogenous layer of the active materials was formed onto glassy carbon with similar loading. As shown in Figure 6A, both catalysts reveal well-defined cathodic peaks in O_2_-saturated solution, confirming the electrocatalytic activity of the synthesized catalysts for ORR. It also can be seen that the C/NiCo_2_O_4_ exhibits more positive peak potential (+0.55 V, vs. Ag/AgCl) with higher cathodic ORR current compared to naked NiCo_2_O_4_ (+0.43 vs. Ag/AgCl). 

In contrast, a featureless signal was observed for C/NiCo_2_O_4_ hybrid in N_2_-saturated solution. From the comparison of the recorded CV signals, the C/NiCo_2_O_4_ composite is more electroactive for ORR than single NiCo_2_O_4_.

To gain further insight into the ORR activity of the as-obtained materials including C/NiCo_2_O_4_, NiCo_2_O_4_, carbon free-NiCo_2_O_4_, and commercial Pt/C, LSVs of different catalysts were performed for a comparative study of the ORR on a rotating-disk electrode (RDE) in O_2_-saturated 0.1 M KOH solution at a rotating speed of 1600 rpm as illustrated in Figure 6B. With respect to the diffusion-limiting current density, C/NiCo_2_O_4_ shows remarkable activity comparable to that of commercial Pt/C (20 wt %) and out performs those of NiCo_2_O_4_ and carbon free-NiCo_2_O_4_. ORR onset potential and half-wave potential (*E*_1/2_) is a key factor to evaluate the kinetics of the reaction and activity of the catalysts. More positive *E*_1/2_ and onset potential confirm an improved activity of the catalyst. It can be seen that the half-wave potential (*E*_1/2_) and onset potential of C/NiCo_2_O_4_ (0.53 V, 0.59 V) is more positive than those of NiCo_2_O_4_ (0.385 V, 0.47 mV), and carbon free-NiCo_2_O_4_ (0.33 V, 0.42 mV).

Clearly, the ORR onset potential of C/NiCo_2_O_4_ hybrid is only about 74 mV more negative compared with that of commercially available Pt/C (20 wt %). In addition, the cathodic current at 0.38 V vs. Ag/AgCl reaches about 5.4 mA cm^−2^, which is a significant when compared to the reported literature [55,56]. The collected onset potential, diffusion-limited current density, and *E*_1/2_ of C/NiCo_2_O_4_ outperform those of many reported transition metal oxides based electrocatalysts as displayed in Table 1.

The excellent ORR activity of the C/NiCo_2_O_4_ is mainly ascribed to these favored features:

(i) Fast electron transport to the catalytically active sites due to improved conductivity.
(ii) Synergetic contact between the carbon matrices and homogeneously distributed Ni and Co species which enhances the accessible active sites and thus lead to better utilization of the electroactive material.
(iii) Richness of electroactive sites can efficiently contribute to the high electrocatalytic activity.
(iv) Well-developed mesoporous structure which can significantly facilitate the diffusion of ions and electrons, adsorption of O_2_-molecules, and subsequently improve the reaction kinetics.

These findings suggest that C/NiCo_2_O_4_ nanofibers is promising ORR electrocatalyst.

Additionally, LSV spectra for C/NiCo_2_O_4_ and commercial Pt/C (20 wt %) were measured under various rotating rates from 400 to 2000 rpm in O_2_-saturated 0.1 M KOH solution and the obtained responses are illustrated in Figure S3A,B. Results show a typical enhancement of the diffusion current density with increasing the rotating rate owing to the improved electrolyte diffusion [62,63].

To analyze the pathways of ORR, the corresponding Koutecky–Levich plots (*j*^−1^ versus ω^−1/2^) were measured and the best linear fit is depicted in Figure S3C,D. Results display a good linearity and close parallelism features, confirm the first-order reaction kinetics with respect to the dissolved O_2_ molecules and similar numbers of electron transferred (*n*) at various potential [64,65]. The number of electron transferred per O_2_-molecules for C/NiCo_2_O_4_ and commercial Pt/C (20 wt %) in the potential range from 0.2 to 0.5 V vs. Ag/AgCl was measured to be 3.87 and 3.94, respectively, indicating that the ORR process at C/NiCo_2_O_4_ catalyst is dominated by a direct four electron pathway (4e^−^) and oxygen molecules were reduced to OH^−^. This finding is significant for non precious electrocatalysts. The enhanced ORR features suggest that the self supported C/NiCo_2_O_4_ nanofibers hold a great potential as a cost-effective alternative to noble metal based electrocatalyst.

**Figure 7:**
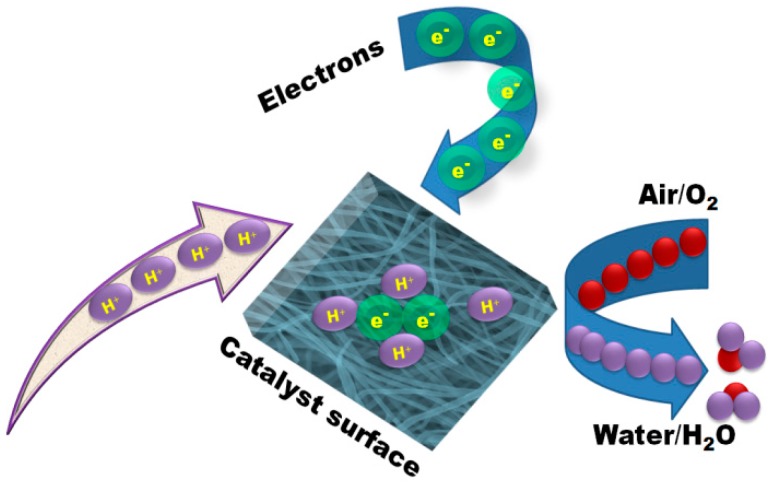
Schematic illustration of the proposed ORR mechanism at the C/NiCo_2_O_4_ catalyst highlights the kinetics of the process and shows that our developed catalyst can efficiently catalyze oxygen molecules via four-electron pathway.

The proposed ORR mechanism for mesoporous C/NiCo_2_O_4_ is graphically illustrated as shown in Figure 7. The preferable porous structure of the as-synthesized catalyst enables a facile adsorption of O_2_ molecules into mesopores and active sites of the catalyst. The metallic species can provide more catalytically active site for electrochemical reduction of O_2_ molecules. In addition, the synergetic effect of the conductive counterparts and the active Co and Ni-species can significantly enhance the ORR performance of the C/NiCo_2_O_4_ catalyst.

The feasible utilization of C/NiCo_2_O_4_ nanofibers as promising candidate in fuel cells technologies can be further illustrated by catalytic selectivity and long term stability. The catalytic selectivity against fuel oxidation is a key factor for efficient ORR electrocatalyst in practical application in fuel cells technologies. Along with this, the immunity against methanol crossover is a crucial issue for potential use.

**Figure 8:**
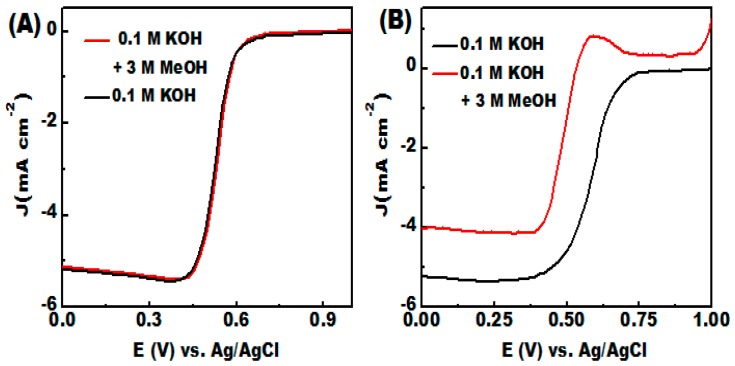
(**A**,**B**) Catalytic selectivity characterization measured in O_2_ saturated 0.1 M KOH solution with the addition of 3 M methanol: (**A**) ORR polarization curves for the as-obtained C/NiCo_2_O_4_ catalyst; and (**B**) ORR polarization responses for the commercially available Pt/C catalyst.

The electrocatalytic selectivity against the electrooxidation of methanol molecules were analyzed by LSV responses in 0.1 M KOH solution with 3 M methanol as given in Figure 8A,B. In presence of methanol, the C/NiCo_2_O_4_ exhibits almost the same *E*_1/2_ with a negligibleloss of current density in case of methanol, indicating a very poor activity towards methanol oxidation (Figure 8A). In contrast, the ORR activity of the commercial Pt/C undergoes a noticeable decay with a drastic negative shift in the *E*_1/2_ compared to that of methanol-free solution. Furthermore, the oxidation of methanol molecules starts at 0.4 V vs. Ag/AgCl with a sharp peak at 0.59 V vs. Ag/AgCl and 160 mV negative shift in the onset potential (Figure 8B). Results indicate that the improved electrocatalytic activity of Pt/C catalyst for methanol electrooxidation can diminish its ORR activity in presence of methanol. These observations clearly indicate that the mesoporous C/NiCo_2_O_4_ catalysts has better tolerance to methanol poisoning.

**Figure 9:**
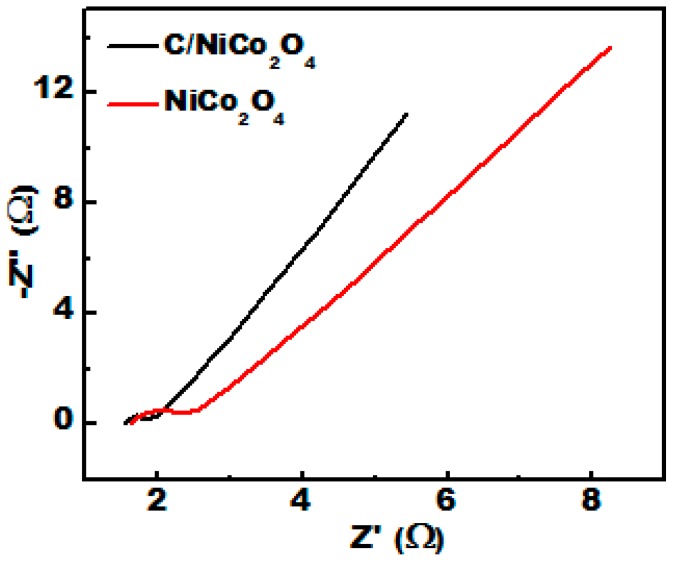
Nyquist plots of C/NiCo_2_O_4_ and NiCo_2_O_4_ nanofibers obtained at room temperature.

To further illustrate the origin of the enhanced electrocatalytic performance of C/NiCo_2_O_4_ nanofibers, electrochemical impedance spectroscopy (EIS) measurements were carried out in the frequency range from 100 kHz to 0.05 Hz with a 5 mV AC perturbation at the open circuit potential. As shown in Figure 9, the Nyquist plots of C/NiCo_2_O_4_ and NiCo_2_O_4_ nanofibers exhibit a depressed semicircle at the high frequency region and straight line at the low frequency region, which ascribed to the charge transfer resistance (Rct) at the electrode/electrolyte interfaces and diffusion process, respectively [66,67]. Clearly, the C/NiCo_2_O_4_ nanofibers present a lower charge transfer resistance (0.27 Ω) than that of single NiCo_2_O_4_ (0.73 Ω), demonstrating faster electron transfer and easy ion accessibility. Moreover, the straight line in the low frequency region of C/NiCo_2_O_4_ displays a slope closer to 90° indicating improved conductivity of the synthesized hybrid.

**Figure 10:**
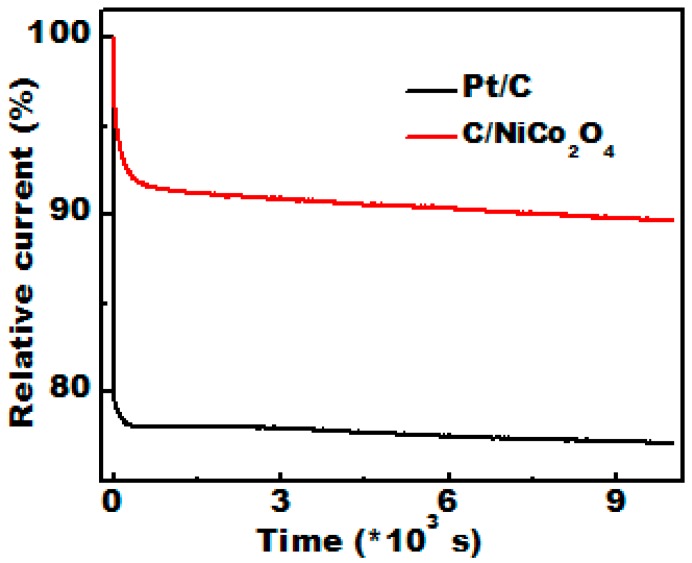
Current–time (i–t) chronoamperometric responses recorded for the hierarchical C/NiCo_2_O_4_ catalyst compared with that of commercial Pt/C catalyst in O_2_ saturated 0.1 M KOH solution for 10,000 s at 0.3 V (vs. Ag/AgCl).

The catalytic stability of electrocatalysts is the most important issue for their practical applications. Thus, the durability of C/NiCo_2_O_4_ compared to commercial Pt/C catalyst was accessed at 0.2 V vs. Ag/AgCl for 10,000 s in O_2_ saturated 0.1 M KOH solution at a rotational speed of 1600 rpm. The obtained current–time (i–t) signals analyzed by chronoamperometry test are shown in Figure 10. As shown, the commercial Pt/C suffered from 22.9% loss in the current density after 10,000 s of continuous operation, whereas the mesoporous C/NiCo_2_O_4_ reveals only 10.4% decrease of the current density. The enhanced electrochemical stability may push NiCo_2_O_4_ nanofibers a potential step forward into practical utilization as high performance electrode material.

## 4. Conclusions

In summary, a facile, one-pot electrospinning technology was utilized to fabricate 1-D C/NiCo_2_O_4_ followed with a carefully intended heat treatment process to form densely packed nanoparticles of NiCo_2_O_4_ conformably encapsulated in highly conductive carbon matrix as an efficient electrocatalyst for ORR. When being employed as a cathode material, the as-prepared porous C/NiCo_2_O_4_ delivered improved ORR properties in terms of cathodic current and onset potential which is a significant improvement compared with single NiCo_2_O_4_ and carbon free NiCo_2_O_4_ catalysts. More importantly, the C/NiCo_2_O_4_ nanofibers reveal a superior electrochemical stability compared to that Pt/C catalyst and achieve up to 89.6% of their initial activity after 10,000 s. The high surface area, accessible electroactive sites, and conductive carbon matrices combined with well-defined mesoporous structure of C/NiCo_2_O_4_ enabled significantly enhanced electrocatalytic activity for ORR.

These results demonstrate that the synthesized C/NiCo_2_O_4_ nanofibers can be investigated as high performance ORR catalyst. The introduced work could be instructive for improving the performance of low conductive nanostructured materials.

## Figures and Tables

**Table 1:** Summary of *E*_1/2_, diffusion-Limited current density (J_L_), and onset potential reported for different electrocatalysts at an electrode rotational speed of 1600 rpm.

Material	Half-Wave Potential (*E*_1/2_, V vs. Ag/AgCl)	Limited Current Density (J_L_) (mA cm^−2^)	Onset Potential (V vs. Ag/AgCl)	Ref
BNC/Co_2_P-2	−0.15	4.85	−0.07	[57]
NiCo_2_O_4_-rGO	about −0.35	2.0	−0.088	[8]
Co(OH)_2_/graphene	about −0.186	0.61	−0.05	[58]
CoO_x_/NCNCs	−0.174	about 5.28	−0.10	[59]
CoCN@CoO_x_(18)/NG	−0.16	5.62	about −0.1	[60]
G–Co/CoO NPs	−0.176	about 4.6	about −0.13	[61]
C/NiCo_2_O_4_	0.59	5.4	0.53	This work

## Supplementary Materials

The following are available online at www.mdpi.com/1996-1944/9/9/759/s1. Figure S1: (A–D) Top-view FE-SEM micrographs of the as-prepared fibers recorded at different locations, (A,B) SEM images of electrospun NiCo_2_O_4_ nanofibers and (C,D) SEM images of electrospun C/NiCo_2_O_4_ hybrid nanofibers; Figure S2: WA-XRD patterns of (a) C/NiCo_2_O_4_ composite nanofibers and (b) NiCo_2_O_4_ nanofibers; Figure S3: (A,B) RDE voltammograms collected in O_2_ saturated 0.1 M KOH solution at various rotational speeds of (A) C/NiCo_2_O_4_catalyst and (B) commercial Pt/C catalyst; (C,D) The corresponding Koutecky–Levich plots derived from the RDE voltammograms of (C) C/NiCo_2_O_4_ hybrid catalyst and (D) commercial Pt/C catalyst.
