# Supplementary material for: Carbon Supported Engineering NiCo2O4 Hybrid Nanofibers with Enhanced Electrocatalytic Activity for Oxygen Reduction Reaction
Source: Materials (Basel). 2016 Sep 6;9(9):759. doi: 10.3390/ma9090759 (PMC5457091; doi:10.3390/ma9090759)
Supplement: Supplementary file 1 [file materials-09-00759-s001.pdf]

Supplementary Materials: Carbon Supported Engineering $NiCo_2O_4$ Hybrid Nanofibers with Enhanced Electrocatalytic Activity for Oxygen Reduction Reaction

Diab Hassan, Sherif El-safty, Khalil Abdelrazek Khalil, Montasser Dewidar and Gamal Abu El-magd

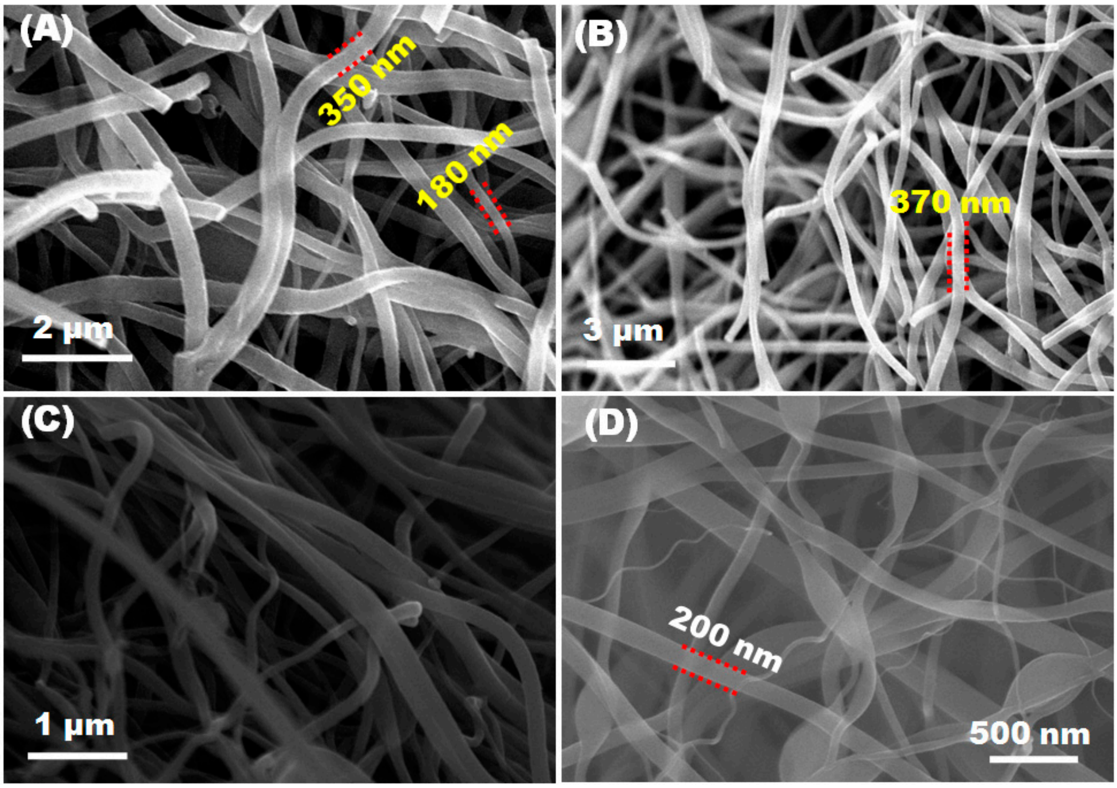

Figure S1. (**A**–**D**) Top-view FE-SEM micrographs of the as-spun fibers recorded at different locations; (**A**,**B**) SEM images of $NiCo_2O_4$ nanofibers; and (**C**,**D**) SEM images of electrospun C/$NiCo_2O_4$ hybrid nanofibers.

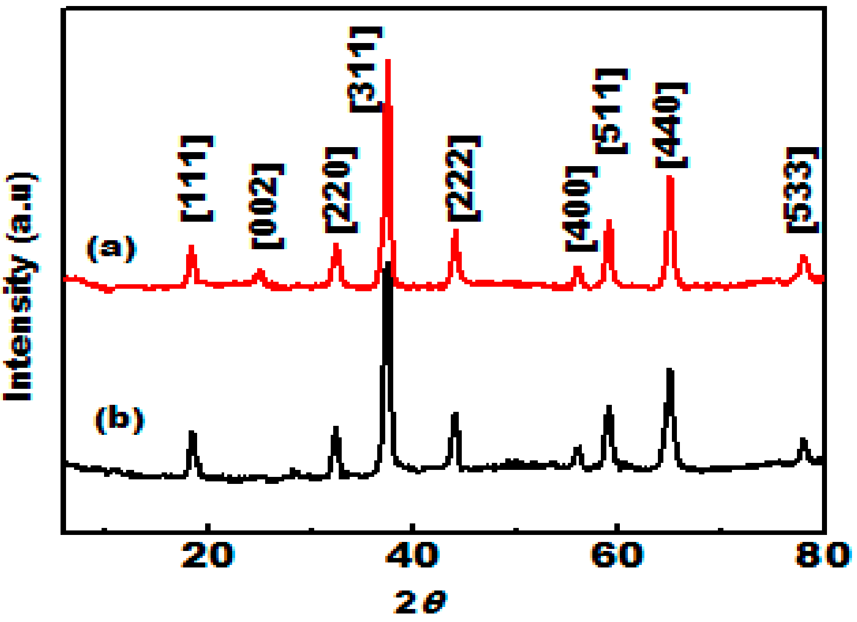

Figure S2. WA-XRD patterns of line a C/$NiCo_2O_4$ composite nanofibers and line b $NiCo_2O_4$ nanofibers.

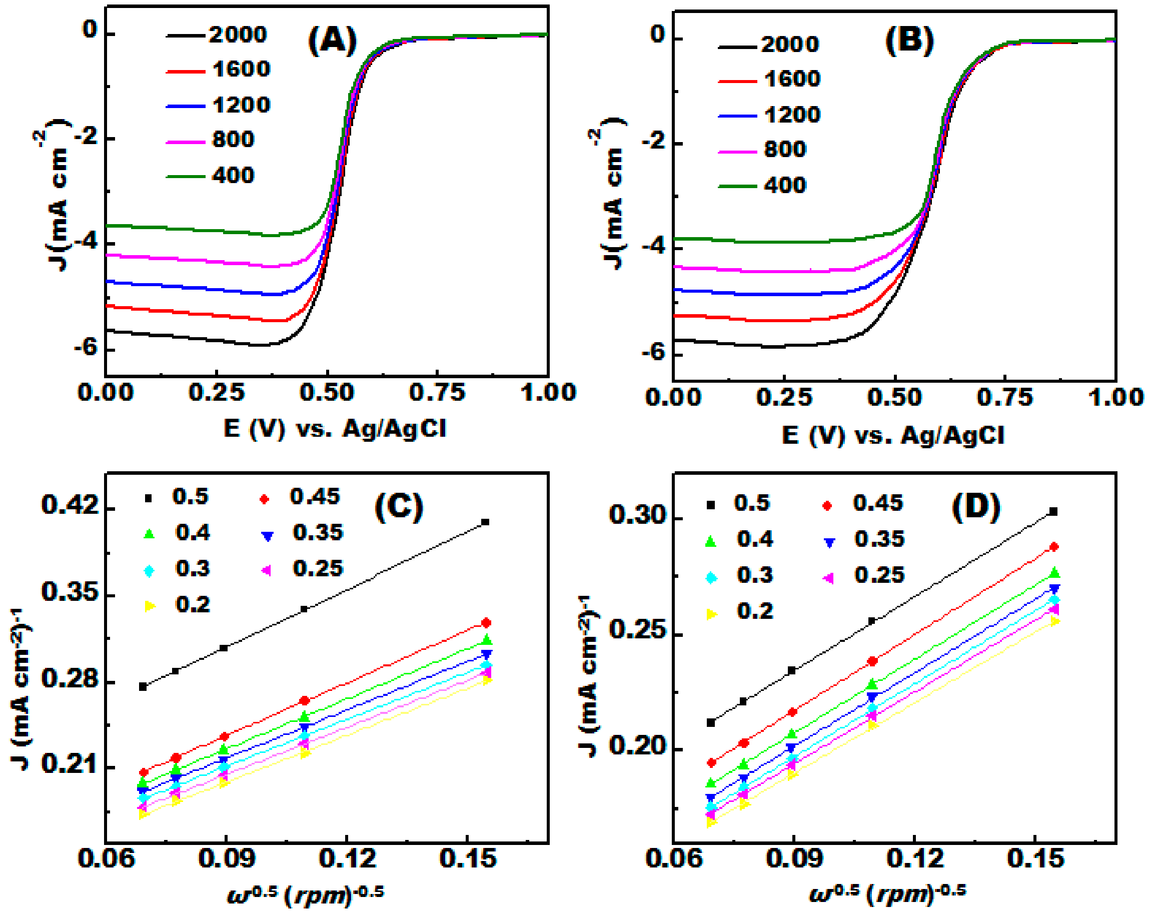

Figure S3. (**A**,**B**) RDE voltammograms collected in O_2 saturated 0.1 M KOH solution at various rotational speeds of (**A**) C/$NiCo_2O_4$ catalyst and (**B**) commercial Pt/C catalyst; (**C**,**D**) The corresponding Koutecky–Levich plots derived from the RDE voltammograms of (**C**) C/$NiCo_2O_4$ hybrid catalyst and (**D**) commercial Pt/C catalyst.
